# Membranous Nephropathy Secondary to Graves’ Disease: A Case Report

**DOI:** 10.3389/fimmu.2022.824124

**Published:** 2022-06-06

**Authors:** Precil Diego Miranda de Menezes Neves, Monique Pereira Rêgo Muniz, Giuliano Ferreira Morgantetti, Érico Murilo Monteiro Cutrim, Carlos de Andrade Macieira, Natalino Salgado-Filho, Joyce Santos Lages, Dyego José de Araújo Brito, Kaile de Araújo Cunha, Giuseppe Cesare Gatto, Gyl Eanes Barros Silva

**Affiliations:** ^1^Nephrology Division, University of São Paulo School of Medicine, São Paulo, Brazil; ^2^Nephrology and Dialysis Center, Oswaldo Cruz German Hospital, São Paulo, Brazil; ^3^Department of Nephrology, Federal University of Maranhão, São Luís, Brazil; ^4^Pathology Division, Ribeirão Preto Medical School, University of São Paulo, Ribeirão Preto, Brazil; ^5^University Hospital of Federal University of Maranhão, São Luís, Brazil; ^6^Nephrology Service, University Hospital, University of Brasília, Brasília, Brazil

**Keywords:** Graves’ disease, membranous nephropathy, auto-immune thyroiditis, kidney biopsy, renal pathology

## Abstract

Membranous nephropathy (MN) is a form of kidney disease that is idiopathic in 70%–80% of cases. Glomerular involvement in autoimmune thyroiditis can occur in 10%–30% of patients, and MN manifests in association with Hashimoto thyroiditis in up to 20% of the cases with glomerular involvement. Reports of MN associated with Graves’ disease (GD) are extremely rare in the current literature. Herein, we report the case of a 46-year-old man admitted to the hospital with nephrotic syndrome and symptomatic hyperthyroidism due to GD. Kidney biopsy revealed a secondary MN pattern. Immunohistochemical staining for PLA2R was negative, and thyroglobulin showed weak and segmental staining along the glomerular capillary. Anti-thyroid peroxidase (TPO) antibody test was not performed. The patient was treated for GD with methimazole and prednisone, and despite reaching clinical improvement after 8 months, proteinuria remained close to nephrotic levels. In this scenario, the patient was submitted to radioactive iodine, and there was a dramatic reduction in proteinuria levels after treatment. In conclusion, GD association with MN is rare, and when present, diagnosis using PLA2R and immunohistochemistry can be useful in determining association. In addition, radioactive iodine therapy can be an effective treatment modality when preceded with immunosuppressive corticosteroid therapy.

## Introduction

Membranous nephropathy (MN) is a glomerulopathy associated with subepithelial immune deposits that induces a spectrum of changes in the glomerular basement membrane (GBM). MN is idiopathic in most cases (70%–80%, primary MN), while the remaining cases are associated with other conditions, ranging from infections, autoimmune diseases, neoplasms, and medications (secondary MN) (1, 2).

Clinical and laboratorial presentation of primary and secondary forms of MN are indistinguishable, with the differential diagnosis relying on an investigation of associated conditions (3). The glomerular involvement in patients with thyroid autoimmune diseases (especially in Hashimoto thyroiditis) can occur in up to 10%–30% of the cases, and MN is present in 20% of these cases. The high prevalence of MN in thyroiditis cases with glomerular involvement suggests a thyroid-associated antigen pathophysiological pathway (4). In contrast to Hashimoto’s disease, Graves’ disease (GD) association with any kind of glomerular disease is extremely rare.

GD is the main cause of hyperthyroidism worldwide. It is a relatively common disease in the overall population and has an established treatment. Only a few cases have reported the association of GD with glomerulopathies. The present study aims to present a case of MN secondary to GD and to review the current literature involving GD and glomerular diseases.

## Case Report

A 46-year-old man with history of hypertension for 4 years and undergoing treatment with Losartan (50 mg/day) and Hydrochlorothiazide (25 mg/day) was referred to the Nephrology Department. He complained of a 3-week history of progressive lower limbs edema evolving to anasarca, fatigue, minimum effort dyspnea, foamy urine, insomnia, and malaise. He had no familial or genetic background of renal diseases.

Upon admission, the patient presented edema, tachypnea, hypertension, and a systolic heart murmur. Previous laboratory tests showed the following: serum creatinine, 1.09 mg/dl; lactic dehydrogenase, 344 U/L; free-T4, 7.63 ng/dl; and thyroid stimulating hormone (TSH), 0.005 mU/L. Serum protein electrophoresis revealed total proteins of 4.0 g/dl and A/G ratio of 0.5, without a serum monoclonal spike. The patient underwent therapy with intravenous furosemide, and the antihypertensive therapy was maintained.

A subsequent laboratory investigation revealed creatinine of 1.09 mg/dl, estimated glomerular filtration rate (by CKD-EPI equation) of 42 ml/min/1.73 m^2^, blood urea nitrogen (BUN) of 35 mg/dl, total proteins of 3.5 g/dl, albumin of 1.3 g/dl, lactate dehydrogenase of 244 U/L, total cholesterol of 170 mg/dl, C-reactive protein, 0.61 mg/dl, hemoglobin of 6.3 g/dl, 24-h proteinuria of 11,131 mg, C3 of 90.9 g/dl, C4 of 19.7 mg/dl, anti-TPO > 600 UI/ml (normal range, <15 UI/ml), anti-TG > 4,000 UI/ml (normal range, <40 UI/ml), free T4 of 72.67 ng/dl (normal range, 0.8 to 1.7 ng/dl), and TSH < 0.005 mUI/L (normal range, 0.3–4.0 mUI/L. Serum markers for HIV, hepatitis B and C, venereal disease research laboratory test (VDRL), lupus (ANA, anti-dsDNA, anti-SM, anti-SSA, and anti-SSB), vasculitis (ANCA), and rheumatoid arthirtis (RF). Urinalysis revealed proteins and granular casts. A thyroid ultrasound showed an ecographic pattern consistent with autoimmune thyroiditis, and an echocardiogram showed no relevant heart abnormalities. GD was diagnosed, and the patient was started on methimazole 20 mg day and prednisone 60 mg/day The treatment of anasarca was based on water and salt restriction plus furosemide.

**Figure 1:**
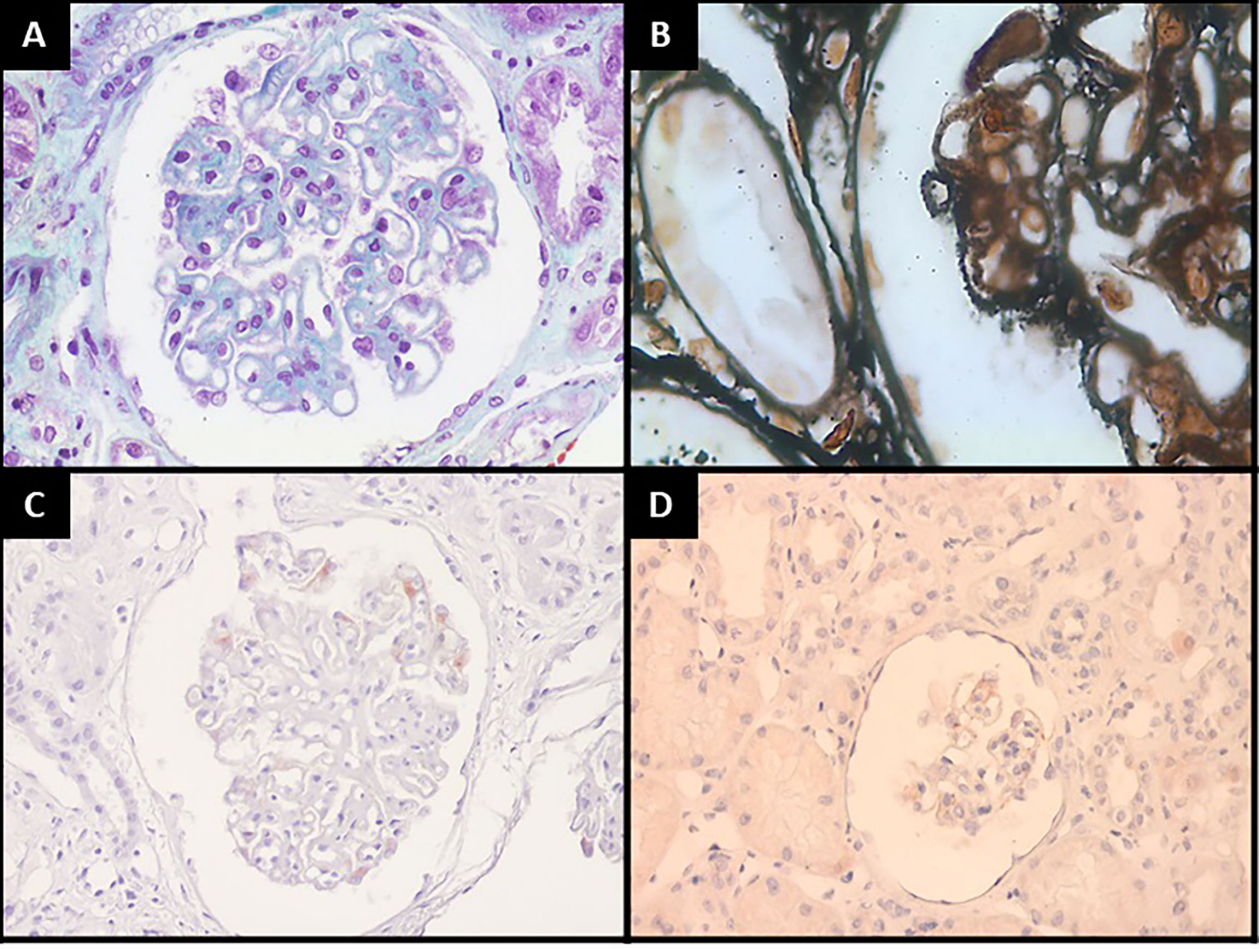
Kidney biopsy findings: **(A)** Global glomerular basement membrane thickening (Masson’s Trichrome, 400×). **(B)** Glomerular characteristic deposits forming “spikes” and “chain links” figures (periodic acid methenamine, 1,000×). **(C)** PLA2R immunohistochemical stain revealing no immunoreactivity (400×). **(D)** Some glomeruli show weak and segmental staining for thyroglobulin along the glomerular capillary (200×).

A renal biopsy was performed to investigate nephrotic syndrome. Light microscopy revealed a diffuse GBM thickening (**Figure 1A**) with characteristic deposits (“spikes” and “chain links”) under silver stain (**Figure 1B**) and a mild mesangial matrix expansion and focal segmental mesangial proliferation. The tubular compartment showed moderate atrophy, and the interstitial compartment showed focal fibrosis. Immunofluorescence microscopy evidenced strong immunoreactivity for IgG (3+/3+), in granular pattern, and global and diffuse distribution along the GBM, in addition to 2+/3+ staining for IgA, IgM, C3, C1q, Kappa resembling IgG, in a “full house” pattern. Immunohistochemical staining for PLA2R was negative (**Figure 1C**) for thyroglobulin (TG) (**Figure 1D**), showing weak and segmental staining along the glomerular capillary, and anti-thyroid peroxidase (TPO) antibody was not available. The association of histological findings were compatible with a stage III membranous nephropathy, and the immunofluorescence pattern suggested a secondary form of the disease.

The patient was then submitted to colonoscopy, upper digestive endoscopy, computed tomography of the thorax, abdomen, and pelvis, and serum analysis of prostate-specific antigen, carcinoembryonic antigen, and alpha fetoprotein to rule out a non-diagnosed cancer as a secondary cause of MN. All tests showed negative results.

After an 8-month treatment regimen with methimazole and corticosteroids, even with normal serum levels of thyroid hormones, nephrotic proteinuria was maintained despite pharmacological treatment of Graves’ disease. In this scenario, the patient underwernt radioiodine thyroid ablation, reaching proteinuria reduction and maintaining stable renal and thyroid parameters since then (**Table 1**). Now, 48 months after thyroid ablation, he currently maintains a serum creatinine of 1.1 mg/dl and 24-h proteinuria of 243 mg.

**Table 1:** Follow-up of patient’s laboratory tests.

	Admission	Pre-ablation	Follow-up	Follow-up
		(after 8 months )	(after 14 months)	(after 48 months)
Creatinine (md/dl)	1.09	1.1	1.4	1.1
Albumin (g/dl)	1.3	3.3	4.2	
TSH (mIU/L)	<0.005	0.01	–	–
Free-T4 (ng/dl)	–	13.23	–	–
Proteinuria (mg/24 h)	11,131	2,977	456	243

## Discussion

Glomerular diseases associated with GD are rarely described in the literature (1, 2). Membranous nephropathy is the most frequent histological finding in these patients (5–8); however, cases of minimal change disease, IgA nephropathy, and membranoproliferative glomerulonephritis have also been reported (9–11). There have also been reported cases of crescent glomerulonephritis (CG) associated with ANCA-positive vasculitis, as a complication of treatment of GD with propylthiouracil, benzilthiouracil, and carbimazole/methimazole (12–14).

Our literature review identified 18 cases of glomerular diseases associated with GD (**Table 2**)with median age of symptom presentation of 33 years ( ± 16 years) and no gender predominance. Currently, two non-mutually mechanisms are proposed to explain the association of MN and GD: (1) *in situ* immune response against TG deposition at the subepithelial level and (2) circulating immune complexes that can be trapped at the subendothelial level due to increased glomerular permeability. None of these proposed mechanisms explain how these immune components would transverse the GBM. Most plausibly, the proteins dissociate to pass through the GBM to reassemble inside the sub-epithelial space (4). It is noteworthy that both mechanisms can be associated with radioiodine therapy. A third possible mechanism would be the “epitope dispersion,” a phenomenon that involves an inflammatory response to a wider set of epitopes of both the target molecule and a cross-reaction with a myriad of different epitopes due to failure to eliminate the given target molecule. Thus, the glomerular immune-mediated lesion would be caused by a subset of antibodies directed to thyroglobulin or thyroid peroxidase, and glomerular antibodies (4).

**Table 2:** Literature review of cases with association of membranous nephropathy and Graves’ disease.

Author	n	Gender	Age	Serum Ab	LM	IM	Atg	Treatment	Diseases associated	Proteinuria follow-up (time)
Ploth et al. (15)	1	M	26	–	MN/MPGN	IgG, IgM, C3	TG	PTU, ¹³¹I	After ¹³¹I	16.5 g/day→0.45 g/day (9 months)
Horvath et al. (16)	1	F	60	ANTI-TG, ANTI-TPO	MN	IgG, IgM, C3	TG	MTZ, Thyroidectomy	–	32 g/day→10 g/day (11 months)
Weetman et al. (17)	5	N/A	23	TRAB	MN	NA	NA	CBZ, Thyroidectomy	GD after MN	14 g/day→3 g/day (15 years)
		NA	19	ANTI-TG, ANTI-TPO	MN	IgG, C3	NA	CBZ, I¹³¹	GD after MN	8.2 g/day→6.4 g/day (11 years)
		NA	23	ANTI-TG	MN	IgG, C3	NA	NA	GD after MN	7.0 g/day→5.8 g/day (15 years)
		NA	33	ANTI-TG, ANTI-TPO, TRAB	MN	IgG, C3	NA	CBZ, TDX, AIE, AZA	GD after MN	8.0 g/day→0.7 g/day (9 years)
		NA	49	ANTI-TPO, TRAB	MN	NA	NA	CBZ	GD after MN	2.2 g/day→2.6 g/day (9 years)
Jordan et al. (5)	1	F	8	ANA, ANTI-TG	MN/MPGN	IgG, IgM, C3	TG	PTU→MTZ→ Partial thyroidectomy, T4→ PRED	–	6.0 g/day→1.1 g/day (NA)
Sato et al. (6)	1	M	58	ANTI-TPO, ANTI-TG, TRAB	MN	IgG, C3	TG	MTZ	–	3.69 g→0.2–2.5 g/day (16 days)
Becker et al. (7)	1	M	26	ANTI-TPO	MN	IgG, IgM, C3, C4, κ, λ, Fbr.	–	¹³¹I, DTP	After ¹³¹I	7.2 g/day→NR (NA)
Grcevska et al. (18)	1	M	25	ANTI-TG	MN	IgG, C3	NA	PTU	–	8.4 g/day→NR (NA)
Shima et al. (19)	1	F	6	ANTI-TPO, ANTI-TG, TRAB	MN	IgG, C3, C1q	TPO	MTZ, ACE	–	4.32 g/day→0.3 g/day (3 months)
Vakrani et al. (8)	1	M	50	ANTI-TPO	MN	IgG, C3	NA	MTZ, ¹³¹I → PRED, DTP, ACE, STN	After ¹³¹I	3.6 g/day→0.25 g/day (3 weeks)
Sasaki et al. (20)	1	F	40	ANTI-TPO, ANTI-TG	MN	IgG, IgM, IgA, C3, Fib	TPO	PTU, MTZ → ¹³¹I	–	4.2 g/gCr→1.24 g/gCr (3 months)
Vanacker et al. (21)	1	F	42	ANA	MN	IgG, IgM, C3, C1q	NA	MTZ→ACE	IgA Deficiency	3,85 g/day→0.58 g/day (NA)
Moniwa et al. (22)	1	F	16	ANTI-TPO, ANTI-TG, TRAB, TSAB	MN	IgG, IgA, C3, κ, λ	TPO	TMZ + ACE	–	3g/g → 0.83g/g (12 months after startinf MTZ)
Cakir et al. (23)	1	F	15	ANTI-TPO, ANTI-TG, TRAB, ANA	MN	IgG, IgM, C3, κ, λ	NA	MTZ → RTX (4 doses with 4 weeks interval)	–	1.9g/m^2^/day → 0.15 g/m^2^/day 3 months after RTX therapy
Presented case	1	M	46	ANTI-TPO, ANTI-TG	MN	IgG, IgM, IgA, C3, C1q, κ, λ	TG	PRED, MTZ → ¹³¹I	–	11.131 g/day→0.243 g/day (48 months)

Ab, antibody; ACEi, angiotensin-converting enzyme inhibitor; Atg, serum antigens; CBZ, carbimazole; F, female; GD, Graves’ disease; ¹³¹I, radioiodine; IF, immunofluorescence microscopy; LM, light microscopy; M, male; MN, membranous nephropathy; MPGN, membranoproliferative glomerulonephritis; MTZ, methimazole; n, number of cases; NA, not available; PRED, prednisone; PTU, propylthiouracil; RTX: Rituximab; STN, statin; T4, levothyroxine; TG, thyroglobulin; TMZ, thiamazol; TPO, thyroperoxidase, TRAB, thyroid-stimulating hormone receptor antibody; TSAB, thyroid-stimulating antibody.

Our presented case revealed a histological pattern suggestive of secondary MN based on the mild mesangial matrix expansion, some degree of segmental mesangial cellular proliferation, immunofluorescence microscopy in a “full house” staining pattern, and a negative PLA2R staining and a weak staining for thyroglobulin, which favor a possible association with thyroid disease antigens. In the setting of a secondary MN, the KDIGO Clinical Practice Guideline for the Management of Glomerular Diseases recommends treatment of the associated disease and no specific treatment of MN. In this case, it is unsure if the use of cortiscoteroids as an adjuvant treatment of GD could produce any effect in treating the glomerular disease *per se*, as the isolated use of this drug is not recommended to treat MN (24).

There is no clear guideline for treating patients with MN associated with GD. It is clear that the patient showed substantial proteinuria improvement after ablation; however, there have been reported cases of secondary GMN development after radioiodine treatment, probably due to the exacerbation of the previously discussed immune pathways, with thyroid tissue destruction and the liberation of a large quantity of thyroid antigens in the bloodstream (7, 8, 15). On the other hand, one of five patients described in a study by Weetman et al. (1981) who used radioiodine did reach any reduction in proteinuria (17). This patient was diagnosed with MN 6 years prior to developing GD, constituting a fact that suggests that the glomerular disease reached an advance stage before treatment or that the association is a correlation that does not necessarily imply causation. A cohort study of patients with GD by Weetman et al. (17) revealed that 14 out of 18 patients treated with radioiodine did not show any sign of proteinuria, while 4 showed some degree of urinary protein. Furthermore, 9 out of the 14 patients without prior proteinuria developed some form in a period of 5–10 weeks after radioiodine treatment. Lastly, three of the four patients in the previous proteinuria group showed a reduction in protein excretion after the same treatment.

We have reported a rare case of MN associated with GD. Further studies researching proteinuria in autoimmune thyroiditis patients can reveal the true prevalence of this association. Radioiodine thyroid ablation seems to be an effective therapeutic option in MN secondary to GD, in association with corticosteroid treatment.

## Data Availability Statement

The original contributions presented in the study are included in the article/supplementary material. Further inquiries can be directed to the corresponding author.

## Ethics Statement

The studies involving human participants were reviewed and approved by Federal University of Maranhão and conducted according to the Declaration of Helsinki. The patients/participants provided their written informed consent to participate in this study. Written informed consent was obtained from the individual for the publication of any potentially identifiable images or data included in this article.

## Author Contributions

PN: acquired data, analyzed data, and wrote the original manuscript. MM: performed clinical care, acquired data, analyzed data, and wrote the original manuscript. GM: perfomed histological analysis, acquired data, analyzed data, and wrote the original manuscript. EC: performed clinical care, acquired data, analyzed data, and wrote the original manuscript. CM: performed clinical care and wrote the original manuscript. NS-F: performed clinical care and wrote the original manuscript. JL: performed clinical care, analyzed data, and wrote the original manuscript. DB: performed clinical care, analyzed data, and wrote the original manuscript. KC: performed clinical care and analyzed data. GG: performed clinical care, analyzed data, and wrote the original manuscript. GG: perfomed histological analysis, acquired data, analyzed data, and wrote the original manuscript. All authors contributed to the article and approved the submitted version.

## Conflict of Interest

The authors declare that the research was conducted in the absence of any commercial or financial relationships that could be construed as a potential conflict of interest.

## Publisher’s Note

All claims expressed in this article are solely those of the authors and do not necessarily represent those of their affiliated organizations, or those of the publisher, the editors and the reviewers. Any product that may be evaluated in this article, or claim that may be made by its manufacturer, is not guaranteed or endorsed by the publisher.
